# Yeast expression of mammalian Onzin and fungal FCR1 suggests ancestral functions of PLAC8 proteins in mitochondrial metabolism and DNA repair

**DOI:** 10.1038/s41598-019-43136-3

**Published:** 2019-04-29

**Authors:** Stefania Daghino, Luigi Di Vietro, Luca Petiti, Elena Martino, Cristina Dallabona, Tiziana Lodi, Silvia Perotto

**Affiliations:** 1 Department of Life Sciences and Systems Biology, University of Torino, Viale Mattioli 25, 10125 Torino, Italy; 2 Italian Institute for Genomic Medicine, via Nizza 52, 10126 Torino, Italy; 3 Department of Chemistry, Life Sciences and Environmental Sustainability, University of Parma, Viale delle Scienze 11/A, 43124 Parma, Italy; 4 Present Address: Department of Biochemistry and Biotechnology, Bayer SAS, centre de recherche “la Dargoire” 14, impasse Pierre Baizet CS 99163, 69263 Lyon, CEDEX 09 France

**Keywords:** Iron, Mitochondria, Fungal genes, DNA mismatch repair, Transcriptomics

## Abstract

The cysteine-rich PLAC8 domain of unknown function occurs in proteins found in most Eukaryotes. PLAC8-proteins play important yet diverse roles in different organisms, such as control of cell proliferation in animals and plants or heavy metal resistance in plants and fungi. Mammalian Onzin can be either pro-proliferative or pro-apoptotic, depending on the cell type, whereas fungal FCR1 confers cadmium tolerance. Despite their different role in different organisms, we hypothesized common ancestral functions linked to the PLAC8 domain. To address this hypothesis, and to investigate the molecular function of the PLAC8 domain, murine Onzin and fungal FCR1 were expressed in the PLAC8-free yeast *Saccharomyces cerevisiae*. The two PLAC8-proteins localized in the nucleus and induced almost identical phenotypes and transcriptional changes when exposed to cadmium stress. Like FCR1, Onzin also reduced DNA damage and increased cadmium tolerance by a DUN1-dependent pathway. Both proteins activated transcription of ancient mitochondrial pathways such as leucine and Fe-S cluster biosynthesis, known to regulate cell proliferation and DNA repair in yeast. These results strongly suggest a common ancestral function of PLAC8 proteins and open new perspectives to understand the role of the PLAC8 domain in the cellular biology of Eukaryotes.

## Introduction

The PLAC8 domain was described for the first time in the protein Onzin, the product of the human Placenta-Specific Gene 8^1^. The same domain was later identified in many Eukaryotes, but its biological role remains elusive because PLAC8 proteins seem to play diverse roles in different organisms and cell types. The mammalian Onzin has been reported as a repressed target of the c-Myc oncoprotein^2^, with pro-proliferative anti-apoptotic effects in many cell types and a role in leukemia^3^, hepatic, pancreatic^4,5^ and colon cancer progression^6,7^, but also in adipocyte growth^8^. The same protein has pro-apoptotic activity in other cell types^9^, indicating that the final effect of Onzin is highly dependent on cell type. In plants, PLAC8 genes are also involved in cell proliferation because mutants are altered in organs size. The tomato Fruit Weight 2.2 (FW2.2) negatively influences fruit size and is downregulated in domesticated species^10^. Similar function has been reported for FW2.2-like genes in other plant species, both dicots^11–14^ and monocots^15,16^.

Another cellular role of PLAC8 proteins in plants and fungi is to increase resistance to heavy metals. First described in *Arabidopsis thaliana*, the Plant Cadmium Resistance (PCR) protein family includes proteins that confer resistance to cadmium or zinc. AtPCR1 was suggested to be - or to be part of- a cadmium transporter because of its localization on the plasma membrane in both *A*. *thaliana* and *S*. *cerevisiae*^17^, while AtPCR2 was associated to zinc transport^18^. Although PLAC8 domain containing genes have been annotated in many fungal genomes (see Mycocosm, https://genome.jgi.doe.gov/programs/fungi/index.jsf), the only PLAC8 genes characterized to date are two Fungal Cadmium Resistance (FCR) genes identified in a metal tolerant isolate of the mycorrhizal ascomycete *Oidiodendron maius*^19,20^, which increased cadmium resistance when expressed in *S*. *cerevisiae*. Unlike plant PCRs, OmFCR1 (hereafter FCR1) is not involved in membrane metal transport and was localized in the *S*. *cerevisiae* nucleus, where it physically interacts with Mlh3p, a key player in meiotic crossing-over and a subunit of the DNA mismatch repair (MMR) complex^19^.

According to Cabreira-Cagliari *et al*.^21^, genes containing the PLAC8-domain represent a unique gene family with three distinct subgroups: Type I genes are found in animals, plants and fungi, whereas Types II and III have only been found in plants. Thus, PLAC8 proteins have likely evolved from a common ancestral protein and an intriguing question is whether, despite their very different roles in different organisms and cell types, a molecular function common to all biological systems can be ascribed to the PLAC8 domain.

To address this question, we have used *S*. *cerevisiae* as a model organism to investigate two small, single-domain PLAC8 proteins from taxonomically distant organisms, the mammalian Onzin from *Mus musculus* and the fungal FCR1 from *O*. *maius*. *S*. *cerevisiae* is a good model system because it has been successfully used to test plant and fungal PLAC8 gene functions by heterologous expression^17–20^. In addition, although *S*. *cerevisiae* lacks genes coding for PLAC8 proteins, the PLAC8 domain can be found in the genome of some basal fungi and other members of Saccharomycotina (see Mycocosm website), thus suggesting that the PLAC8 domain appeared early in fungal evolution. Thus, although *S*. *cerevisiae* lacks genes coding for PLAC8 proteins, it likely features the PLAC8-interacting metabolic framework.

When expressed in cadmium-exposed *S*. *cerevisiae*, Onzin and FCR1 displayed very similar phenotypes, as they both conferred cadmium resistance and increased cell proliferation and vitality. Both proteins localized in the yeast nucleus, reduced the mutation frequency in homonucleotide runs and induced similar transcriptomic changes in cells exposed to cadmium. In particular, both PLAC8 proteins up-regulated ancient and conserved metabolic pathways that link mitochondrial functions related to leucine biosynthesis, Fe-S cluster biogenesis and maintenance of nuclear DNA integrity. Although the exact role of the PLAC8 domain remains unclear, our findings provide strong support to the hypothesis of a common Dun1p-dependent ancestral function for this protein domain.

## Results and Discussion

### Both PLAC8 proteins increase growth and survival of yeast cells exposed to cadmium

**Figure 1:**
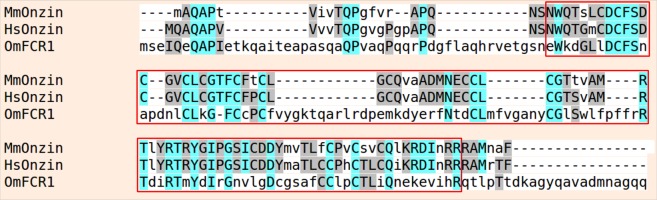
Amino acid sequence alignment of PLAC8 domain-containing proteins. The complete sequences of MmOnzin (from *Mus musculus*), HsOnzin (from *Homo sapiens*) and OmFCR1 (from *Oidiodendron maius*) have been aligned. Similar residues are colored as the most conserved one according to BLOSUM62 average scores: Max: 3.0 (light blue), Low: 0.5 (grey). Lower case non-colored letters indicate amino acid residues with no similarities. The protein alignment was performed using the Phylogeny.fr platform. The red box shows the PLAC8 domain.

Onzin is a small protein highly conserved in all vertebrates and in particular in mammals, where mouse and human orthologous proteins are 83% identical (Fig. 1). By contrast, Onzin shared only 29 amino acids with the fungal PLAC8 protein FCR1, with an overall 25% sequence identity mainly confined to the signature cysteine-rich motif of the PLAC8 domain (Fig. 1).

**Figure 2:**
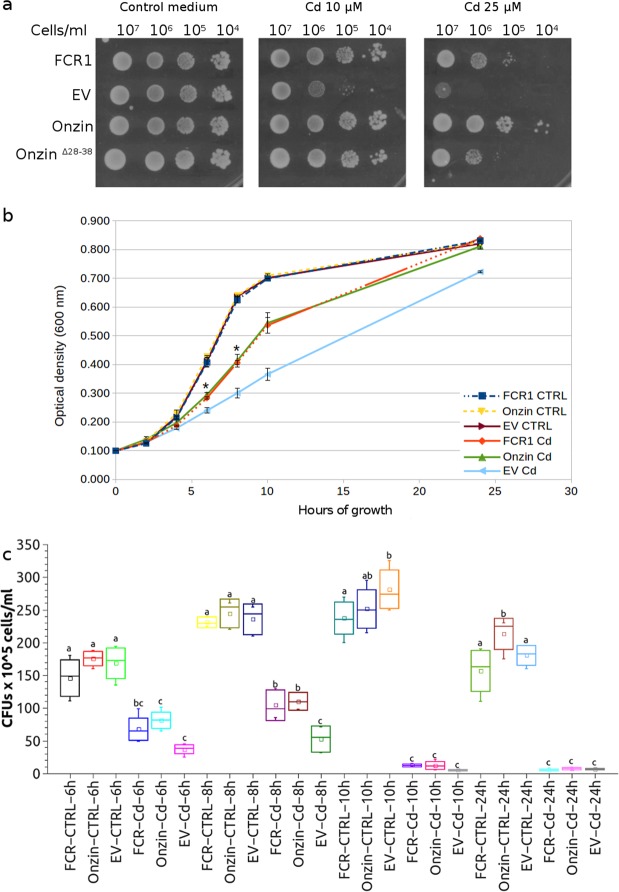
Growth and viability of yeast cells expressing FCR1 and Onzin on cadmium-containing media. (**a**) Spot dilution assay of yeast (EAY1269 strain) expressing FCR1, wild-type Onzin, the truncated Onzin^Δ28–38^ or the empty vector pFL61 (EV). Strains were plated in ten-fold serial dilutions onto YNB-D medium amended with CdSO_4_ (10 or 25 µM) or not (control medium). (**b**) Growth curves of yeast cell cultures in control medium (CTRL) or in a medium containing 25 µM CdSO_4_ (Cd). The optical density (OD_600_) of cultures expressing FCR1, Onzin and pFL61 (EV) was measured after 2-4-6-8-10-24 hours of incubation at 30 °C and 150 rpm. The asterisks indicate time points with a significant difference (n = 3, Shapiro Wilk as normality test, ANOVA with Tukey P < 0.01) between cells expressing FCR1, or Onzin, and cells transformed with the empty vector (EV). (**c**) Cell viability of yeasts expressing FCR1, Onzin or the empty vector pFL61 (EV) after growth for 6-8-10-24 hours in control medium (CTRL) or in a medium containing 25 µM CdSO_4_ (Cd). Colony Forming Units (CFUs) were counted for each yeast culture. Samples showing statistically different CFU numbers (P < 0.05 by ANOVA with Tukey as post-hoc test, n = 6 for 6-10-24 h time points, n = 3 for the 8 h time point, Shapiro Wilk as normality test) are indicated by different letters. The square symbol indicates the mean value. The wiskers indicate the minimum and the maximum values. The top and the bottom of the rectangle indicate ± standard deviation, while the central line of the rectangle indicates the 50%.

A first set of experiments was aimed to compare the phenotype of Onzin expressing *S*. *cerevisiae* exposed to cadmium with the one previously described for FCR1^19^. Spot dilution assays at two CdSO_4_ concentrations (Fig. 2a) showed that both Onzin and FCR1 confer cadmium tolerance to *S*. *cerevisiae*, when compared with the control strain transformed with the empty vector. At 25 μM CdSO_4_, Onzin-expressing yeast grew even better than the FCR1-expressing strain (Fig. 2a). A truncated Onzin lacking the conserved N-terminal region of the PLAC8 domain (Onzin^Δ28–38^) led to a partial loss-of-function phenotype (Fig. 2a), suggesting that this protein region is important for Onzin function. Evaluation of the cadmium half inhibitory concentration (IC50) on the same yeast strains grown in liquid culture yielded fully consistent results (Fig. S1).

Yeast growth and viability was also monitored for 24 h in liquid culture on control medium and on medium containing 25 μM CdSO_4_. On control medium, all yeasts strains showed identical growth curves, as measured by OD_600_, whereas Onzin and FCR1 expressing yeasts grew more than the control strain on cadmium-amended medium, starting from 6 h incubation (Fig. 2b). Cell survival in the same growth experiment was measured by colony forming units (CFUs) count and was similar for all yeast strains grown on control medium. By contrast, Onzin and FCR1 expression led to CFU numbers higher than the empty vector after 6 and 8 h of cadmium exposure (Fig. 2c). At later time points, cell survival on cadmium-amended medium was low for all yeast strains (Fig. 2c). Thus, Onzin expression conferred to yeast a cadmium-tolerant phenotype similar to FCR1^19^. Both FCR1 and Onzin expression increased yeast growth exclusively on cadmium-containing medium, indicating a protein function only measurable during cadmium stress.

### Both PLAC8 proteins localize to the yeast nucleus

**Figure 3:**
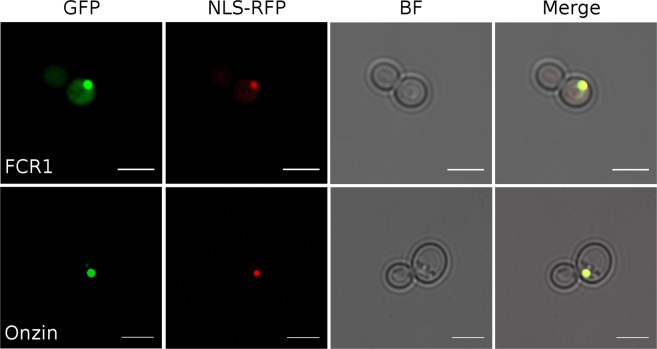
Subcellular localization of FCR1 and Onzin proteins in yeast cells. The FCR1-EGFP and the Onzin-EGFP fusion proteins (GFP) were localized to the yeast nucleus, as indicated by co-localization (Merge) with a fusion protein carrying a nuclear localization signal (NLS)-RFP. BF: bright field image of the yeast cells. Scale bar is 5 μm.

Subcellular localization (Fig. 3) in yeast cells by C-terminal tagging with the Enhanced Green Fluorescent Protein (EGFP) showed that both Onzin and FCR1 co-localized with the Red Fluorescent Protein fused to the nuclear localization signal.

Although there is currently no information on PLAC8 protein localization in fungi (e.g. FCR1 in *O*. *maius*), PLAC8 proteins have been localized in plant and mammalian cells. In plants, PCR proteins have been found to associate to the plasma membrane, where they regulate metal transport^17,18^. More complex is the localization of PLAC8 proteins in mammalian cells, where Onzin in particular has been observed in the nucleus^22^, in the cytoplasm^7,23^, in the lysosomal compartment^24^ or associated to the plasma membrane^4,25^. Interestingly, Onzin could be dynamically redistributed between the nucleus, the cytoplasm and the plasma membrane depending on the growth conditions and the relative abundance of interacting proteins^2^. Thus, the nuclear localization of EGFP-tagged Onzin in yeast is not in contrast with observations in mammalian cells. Moreover, the nuclear localization of FCR1 and Onzin would exclude a direct role in metal detoxification based on membrane transport, a possibility already excluded for FCR1 in previous studies^19^. We therefore investigated possible functions of the two PLAC8 proteins correlated with their nuclear localization.

### Both PLAC8 proteins physically interact with Mlh3p and reduce cadmium induced DNA mutations

Cadmium does not damage DNA directly but is mutagenic because it interferes with the cellular response to DNA damage^26^ and inhibits all major DNA repair pathways, including the mismatch repair (MMR) complex^26–28^. FCR1 was found to physically interact with Mlh3p, a component of the MMR complex^19^ and a yeast two hybrid assay confirmed its interaction with the C-terminal region of both the yeast and the *O*. *maius* Mlh3p (Supplementary Fig. S2). The same assay revealed a similar, albeit weaker, interaction of Onzin with the C-terminal region of the yeast and the mouse Mlh3 proteins (Supplementary Fig. S2).

Previous experiments using the forward mutation assay at the canavanine-resistance (*CAN1*) locus did not reveal an influence of FCR1 on the DNA mutation rate under cadmium stress^19^. However, the *CAN1* assay could reveal only small differences between wild-type and a *mlh3*-defective yeast^29^. Here, we investigated the influence of FCR1 and Onzin expression on cadmium-induced mutagenicity with the more sensitive yeast *lys2*::*insE-A*_14_ reversion assay, based on the restoration of the open reading frame in a mononucleotide run of 10 adenines within the lys2::insE-A_14_ allele^30^. In cells defective of the MMR system, this assay could reveal 10- to 1,000-folds increase in mutations^31^. On control medium, yeast strains transformed with the empty vector or with the two PLAC8 genes showed no differences in DNA mutation rate (Table 1). As expected, cadmium exposure increased the DNA mutation rate, but expression of both FCR1 and Onzin reduced cadmium-induced DNA mutagenesis about three-folds, when compared with the empty vector (Table 1). FCR1 and Onzin physically interact with Mlh3p, and a *mlh3* defective mutant was used to investigate whether reduction in the mutation rate required this protein. As expected^28^, the *mlh3* strain transformed with the empty vector showed a higher background mutation rate both in control medium and in cadmium-amended medium, but expression of FCR1 and Onzin led to a five-fold reduction in the DNA mutation rate when this mutant was exposed to cadmium (Table 1). Thus, cells exposed to cadmium display a reduced DNA mutation rate when they express either FCR1 or Onzin, although the relationship between these two PLAC8 proteins and the MMR complex remains unclear.

**Table 1:** Influence of Onzin and FCR1 on DNA mutation rate measured by the Lys2::insE-A_14_ reversion assay.

Strain	Vector	Treatment	Reversion Rate × 10^−5^ (confidence intervals)	Fold decrease in mutation rate^a^	n^b^
WT	pFL61-EV	Control medium	0,14 (0,30 ÷ 0,09)	1	30
	pFL61-FCR1		0,19 (0,21 ÷ 0,13)	0,74	20
	pFL61-Onzin		0,18 (0,20 ÷ 0,16)	0,74	20
WT	pFL61-EV	CdSO_4_ 1 μM	5,49 (6,10 ÷ 5,00)	1	30
	pFL61-FCR1		1,75 (1,88 ÷ 1,54)	3,14	20
	pFL61-Onzin		1,92 (1,99 ÷ 1,70)	2,85	20
mlh3	pFL61-EV	Control medium	1,55 (1,88 ÷ 1,03)	1	25
	pFL61-FCR1		1,63 (1,80 ÷ 1,20)	0,95	25
	pFL61-Onzin		1,57 (1,68 ÷ 1,04)	0,99	25
mlh3	pFL61-EV	CdSO_4_ 1 μM	14,61 (15,11 ÷ 14,01)	1	25
	pFL61-FCR1		2,8 (3,01 ÷ 2,03)	5,21	25
	pFL61-Onzin		2,55 (2,97 ÷ 2,12)	5,72	25

Reversion to Lys^+^ phenotype was assessed in the wild-type (WT) and *mlh3* yeast mutant strains expressing FCR1, Onzin or the empty vector (EV) on both control medium and cadmium-containing medium. ‘n’ column indicates the number of independent cultures tested from at least two independently constructed strains. Median mutation rates are presented as ×10^−5^ with 95% confidence intervals. Relative mutation rates are compared with the empty vector of the same genetic background strain. ^a^Each median reversion rate was normalized to the empty vector median rate to calculate the fold decrease; ^b^n = number of independent replicates.

### Onzin and FCR1 induce similar transcriptomic changes in cadmium-exposed yeast

To identify the pathways transcriptionally regulated in yeast by the two PLAC8 proteins, an RNAseq experiment was performed after 8 h of exposure to 25 μM CdSO_4_. The diagram in Supplementary Fig. S3 reports the number of transcripts significantly regulated in yeast expressing either FCR1 or Onzin, as compared with the empty vector (log_2_ fold-change threshold >1 or <−1, adjusted p-value < 0.05). Genes up-regulated by both proteins, indicated as *PLAC8 up-regulated* genes, represented 68% and 70% of the total number of yeast genes up-regulated by FCR1 and Onzin, respectively. Genes down-regulated by both proteins, indicated as *PLAC8 down-regulated* genes, represented 59% and 72% of the total number of genes down-regulated by FCR1 and Onzin, respectively. A complete list of FCR1 and Onzin regulated genes is available in the Supplementary Table S1.

“Mitochondrion” and “mitochondrial parts” were the only enriched cellular compartments identified by Gene Ontology (GO) among *PLAC8 up-regulated* genes, together with biological processes related to the biosynthesis of branched-chain amino acids and molecular functions related to metal ion binding, in particular iron (Supplementary Table S2). Consistently with these GO data, *PLAC8 up-regulated* genes were involved in several mitochondrial pathways. “Plasma membrane” was the cellular compartment enriched in *PLAC8 down-regulated* genes, together with molecular functions such as ions, amino acids and sugars transport, and iron homeostasis (Supplementary Table S2).

### Onzin and FCR1 do not activate antioxidant responses to cadmium

Although cadmium is unable to generate free radicals directly, cadmium exposure induces the production of reactive oxygen species (ROS). Activation of antioxidative enzymes and metabolites is therefore a common cell response that could reduce cadmium toxicity^32^. However, none of the *PLAC8 up-regulated* genes in our transcriptomic analysis (Supplementary Table S1) coded for proteins involved in ROS scavenging (e.g. superoxide dismutases or enzymes involved in glutathione metabolism). These data confirm previous experiments suggesting that FCR1 does not confer cadmium tolerance by increasing the antioxidative cell potential^19^.

The most *PLAC8 up-regulated* gene was *ALD5*, coding for a mitochondrial K^+^-activated aldehyde dehydrogenase (ALDH). In yeast, ALDHs have a distinct role in the antioxidant cell responses because they maintain redox balance by supplying reducing equivalents in the form of NADH and NADPH^33^. However, Ald5p seems to play only a minor role as ALDH, because an *ald5* mutant retained 80% of K^+^-activated ALDH activity^34^. ALD4, the major K^+^-activated mitochondrial ALDH, as well as the cytosolic ALD6, were both *PLAC8 down-regulated* genes (Supplementary Table S1). Kurita & Nishida^34^ showed a more important role of mitochondrial Ald5p in the regulation or the biosynthesis of electron transport chain components. We therefore measured total cellular respiration in the yeast strain W303-1B transformed with Onzin, FCR1 or the empty vector. The results (Supplementary Fig. S4) indicate that the overall oxygen consumption was slightly increased (about 10%) in the PLAC8-expressing strains, irrespective of CdSO_4_ exposure. Overall, the transcriptomic data suggest that the two PLAC8 proteins did not reduce cadmium toxicity and mutagenicity simply by increasing the cell antioxidative response.

### Both PLAC8 proteins induce iron-dependent pathways for leucine and iron-sulfur cluster biosynthesis

**Figure 4:**
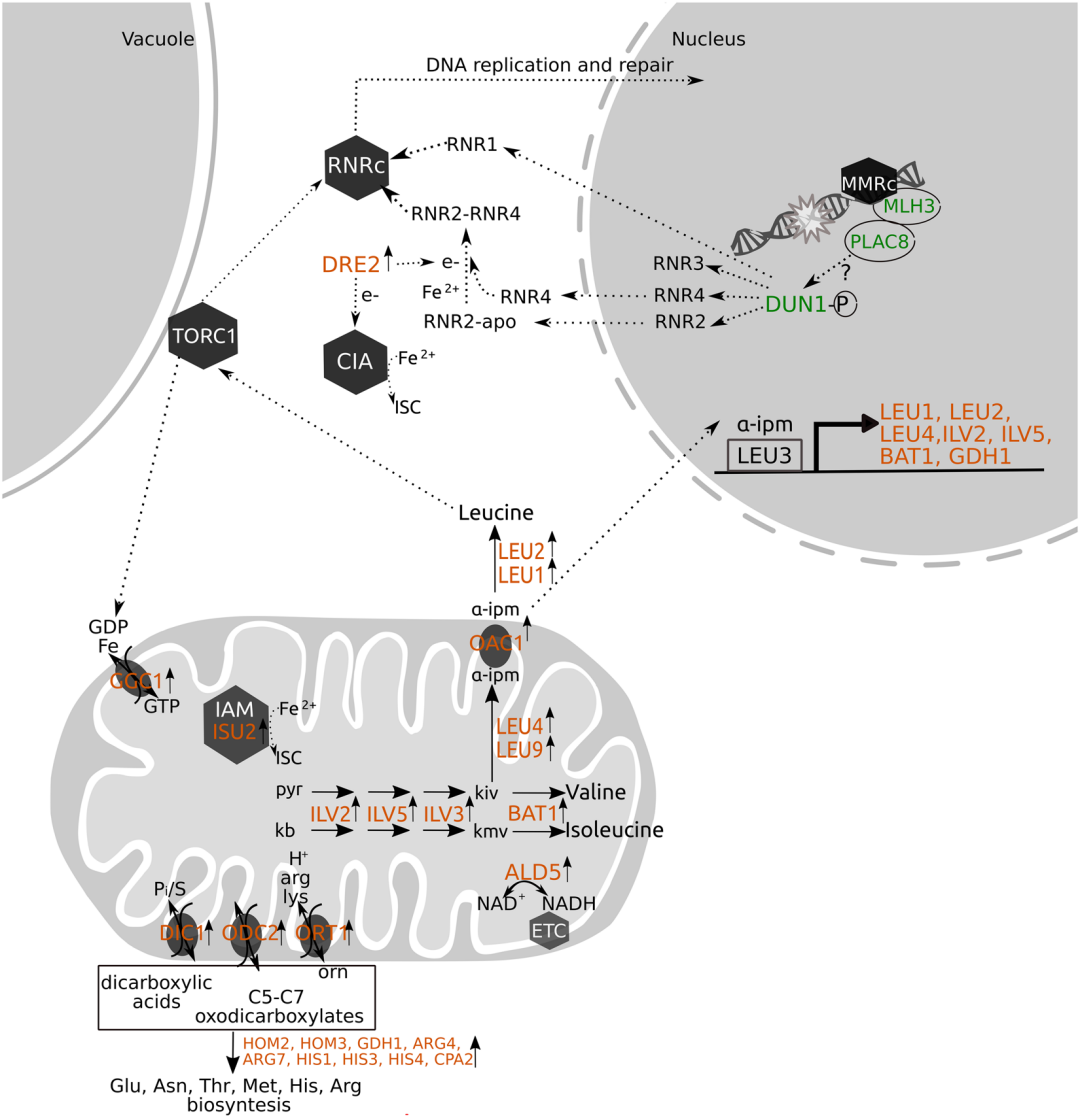
Schematic representation of the cellular functions of the PLAC8 up-regulated genes. PLAC8 up-regulated genes are indicated in orange. Genes for which functional assays were performed are colored in green. Reactions catalyzed by proteins encoded by PLAC8 up-regulated genes are indicated as full arrows. Dotted arrows represent processes or regulatory pathways known from the literature (see text for references). Hexagons represent enzymatic complexes, whereas gray ovals represent membrane carriers. ALD5: aldehyde dehydrogenase 5, BAT1: mitochondrial branched-chain amino acid (BCAA) aminotransferase, CIA: cytosolic ISC assembly, DIC1: dicarboxylate carrier 1, DRE2: Fe-S-containing protein supplying reducing equivalents to the early steps of the cytosolic Fe-S assembly (CIA) pathway, DUN1-P: DNA-damage uninducible kinase, in the phosphorilated active form, ETC: electron transfer chain, GDH1: NADP(+)-dependent glutamate dehydrogenase, GGC1: GDP/GTP carrier 1, IAM: mitochondrial ISC assembly machinery, ILV2: acetolactate synthase, ILV3: dihydroxyacid dehydratase, ILV5: acetohydroxyacid reductoisomerase, ISU2: mitochondrial protein required for iron-sulfur protein synthesis, α-IPM: α-isopropylmalate, pyr: pyruvate, kb: α-ketobutanoate, kiv: α-ketoisovalerate, kmv: a-ketomethylvalerate, LEU1: isopropyl malate isomerase, LEU2: β-IPM dehydrogenase, LEU3: leucine biosynthesis transcription factor, acts as an activator in the presence of α-isopropylmalate, LEU4: α-isopropylmalate synthase, LEU9: α-isopropylmalate synthase (paralog of LEU4), MMR: DNA mismatch repair complex, OAC1: oxaloacetate carrier 1, ODC2: oxodicarboxylate carrier 2, ORT1: ornithine transporter 1, RNRc: ribonucleotide reductases complex, TORC1: *t*arget *o*f *r*apamycin complex 1.

The *PLAC8 up-regulated* genes included key genes involved in amino acid biosynthesis, such as arginine, histidine, methionine and threonine (Supplementary Table S1), but one of the most represented pathways was the super-pathway of leucine, isoleucine, and valine biosynthesis (Supplementary Table S2, Fig. 4). *PLAC8 up-regulated* genes included *ILV2*, *ILV3*, *ILV5* and *BAT1*, involved in common reactions of branched-chain amino acids (BCAAs) biosynthesis, and genes specific for the leucine pathway (*LEU1*, *LEU2*, *LEU4*, *LEU9* and *OAC1*). Oac1p, a mitochondrial oxaloacetate transporter, catalyzes the export to the cytoplasm of α-isopropylmalate, an intermediate of leucine biosynthesis produced inside the mitochondrion^35^. Together with *GDH1*, encoding a major enzyme for ammonia assimilation in *S*. *cerevisiae*, all these *PLAC8 up-regulated* genes are established or potential members of the Leu3p regulon^36^, which is transcriptionally regulated by Leu3p. Leucine is one of the most conserved and potent TORC1 (*target of rapamycin complex* 1) activating growth signals (Fig. 4). TORC1 is a protein complex, conserved throughout Eukaryotes, that functions as a master regulator of cell proliferation, survival and growth^37^.

Two *PLAC8 up-regulated* genes of the Leu3p regulon, the acetohydroxyacid reductoisomerase *ILV5* and the BCAA aminotransferase *BAT1* have an additional transcriptional control by Tpk1p, a subunit of yeast protein kinase A, thought to have a role in controlling mitochondrial iron homeostasis^38^.

The mitochondrion plays a focal role in iron metabolism because is a major generator of heme and Iron-Sulfur Cluster (ISC) cofactors^39^. ISC are among the most ancient and versatile cofactors of proteins involved in many cellular processes such as respiration, DNA synthesis and repair, metabolite biosynthesis, and oxygen transport catalysis^40–43^. Biogenesis of the ISC in Eukaryotes is a highly conserved process that involves the mitochondrial ISC assembly machinery (IAM in Fig. 4), an export system from the mitochondrion and the cytosolic ISC assembly (CIA) machinery, required for cytoplasmic and nuclear ISC-containing enzymes^42^. *PLAC8 up-regulated* genes included *ISU2*^44^ and *DRE2*^45^, coding for essential proteins in the mitochondrial and the cytosolic ISC assembly machineries, respectively (Supplementary Table S1, Fig. 4).

Notably, mitochondrial ISC synthesis in yeast is tightly regulated by the leucine biosynthetic pathway^46^. Leu1p in particular is an abundant cytoplasmic ISC-containing enzyme^46^ and a key regulator of the mitochondrial-cytoplasmic ISC balance^47^. Leu1p activity is also used as a marker of ISC enzyme biogenesis^48^. Leu1p shares high homology to the iron regulatory protein Irp1 in mammalian cells, suggesting an influence on iron metabolism within the cell^49^. Iron deficiency is thought to influence the pathway of leucine biosynthesis by reducing the activities of multiple ISC-containing enzymes, including Leu1p and Ilv3p^50^.

Overall, the transcriptomic data clearly showed that cadmium-exposed yeast cells expressing PLAC8 proteins up-regulated leucine and ISC biosynthesis, two iron-dependent pathways that involve the mitochondrion.

### Expression of both PLAC8 proteins in yeast does not modify intracellular iron content

A correlation between iron homeostasis and cadmium response has been demonstrated by genome-wide screening of *S*. *cerevisiae* deletion mutant collections because many cadmium-sensitive mutants were affected in genes related to iron homeostasis^51,52^. Cadmium interferes with iron homeostasis by reducing iron uptake, and iron addition can rescue cadmium-sensitivity of yeast mutants^51^ and increase cadmium tolerance of *S*. *cerevisiae*^53^. Moreover, cadmium exposure was found to stimulate the expression of several yeast genes related to iron uptake^54^.

The “iron regulon” comprises ca. 30 genes mostly involved in iron acquisition, activated upon iron deficiency by the iron-sensing transcription factors Aft1p and Aft2p^50^. Several *PLAC8 down-regulated* genes (Supplementary Table S1) are known members of the yeast iron regulon, like the high affinity iron uptake system (*FET3* and *FTR1*), components of the siderophore transport system (*FIT2* and *FIT3*, *SIT1*, alias *ARN3*), and the mRNA-binding protein *TIS11* (alias *CTH2*). Cadmium-exposed yeast cells transformed with the empty vector up-regulated all these genes, thus suggesting that they experience a condition of iron depletion, as compared with PLAC8-expressing yeasts. To verify whether FCR1 and Onzin conferred cadmium tolerance by increasing the amount of intracellular iron, we measured total iron content in yeast cells exposed to cadmium for 8 h to 25 μM CdSO_4_, the same conditions used for the transcriptomic experiment. Irrespective of the intracellular Cd-concentration, we found no statistical differences in total iron content of yeast cells expressing FCR1, Onzin or the empty vector (Supplementary Table S3). Although it is still possible that different cell compartments may experience different iron concentrations, these results indicate that down-regulation of the iron regulon in the PLAC8-expressing yeasts does not simply reflect increased iron content. Down-regulation of the iron regulon is a consequence of dissociation from target DNA of the transcriptional activator Aft1p^55^. Interestingly, ISC have been involved in the dissociation of Aft1p from its target promoters under iron sufficiency in *S*. *cerevisiae*^55^. Chen and colleagues^56^ also demonstrated that inhibition of ISC biosynthesis induced the iron regulon in spite of high cytosolic iron levels. Thus, we may speculate that the up-regulated expression of ISC biosynthetic genes in the two PLAC8-expressing yeast strains may down-regulate transcription of the iron regulon.

### The cadmium tolerant PLAC8 phenotype requires Dun1p

Although the phenotype of Onzin and FCR1-expressing yeast exposed to cadmium included increased growth (Fig. 2) and reduced DNA mutation rate (Table 1), no genes specifically related to cell cycle control or DNA damage repair could be identified among the *PLAC8-regulated* transcripts. Of course, we cannot exclude a post-transcriptional regulation of these pathways, but our transcriptomic data suggest other possible scenarios.

Several proteins involved in DNA synthesis and repair contain ISC cofactors, including replicative DNA polymerases and primase, DNA helicases, nucleases, glycoylases and demethylases^57,58^. An interesting mechanism proposed by Arnold and colleagues^59^, named *DNA charge transport*, suggests that DNA processing enzymes containing the ISC cofactor may use electrons released from redox active iron to rapidly and efficiently scan DNA over long molecular distances for mismatches and damages. It is therefore not surprising that impairments in the mitochondrial or the cytosolic ISC assembly machineries are connected with nuclear genomic instability^60,61^. We could speculate that yeast cells expressing PLAC8 proteins may increase their survival because, by up-regulating the ISC biosynthetic machinery, they provide enzymes involved in DNA damage repair with essential cofactors. These active enzymes would more effectively reduce the high DNA mutation rate caused by cadmium (Table 1). Interestingly, Mms19, a late-acting CIA component likely implicated in the delivery of ISC into nuclear and cytosolic apoproteins^58^, was found to be necessary to *Schizosaccharomyces pombe* to grow on cadmium^62^.

Experimental evidence supports specific relationships between ISC biosynthesis and DNA damage repair. Nuclear DNA damage activates at least two different signalling pathways that converge at Dun1p, a protein kinase that controls the DNA damage response in yeast^63^. Dun1p then activates ribonucleotide reductases at multiple levels^58^, thus leading to increased biosynthesis of dNTPs, needed for DNA repair^64^. Dysfunctions in ISC-targeting factors, which are not required for the biogenesis of ISC but act specifically for transferring ISC to mitochondrial target apoproteins, activate a DNA damage checkpoint mediated by the Mec1p–Chk1p–Dun1p signalling transduction pathway^65^. By contrast, dysfunctions of the core mitochondrial ISC assembly machinery induce a second pathway involving a Mec1p-independent activation of Dun1p^64,65^.

**Figure 5:**
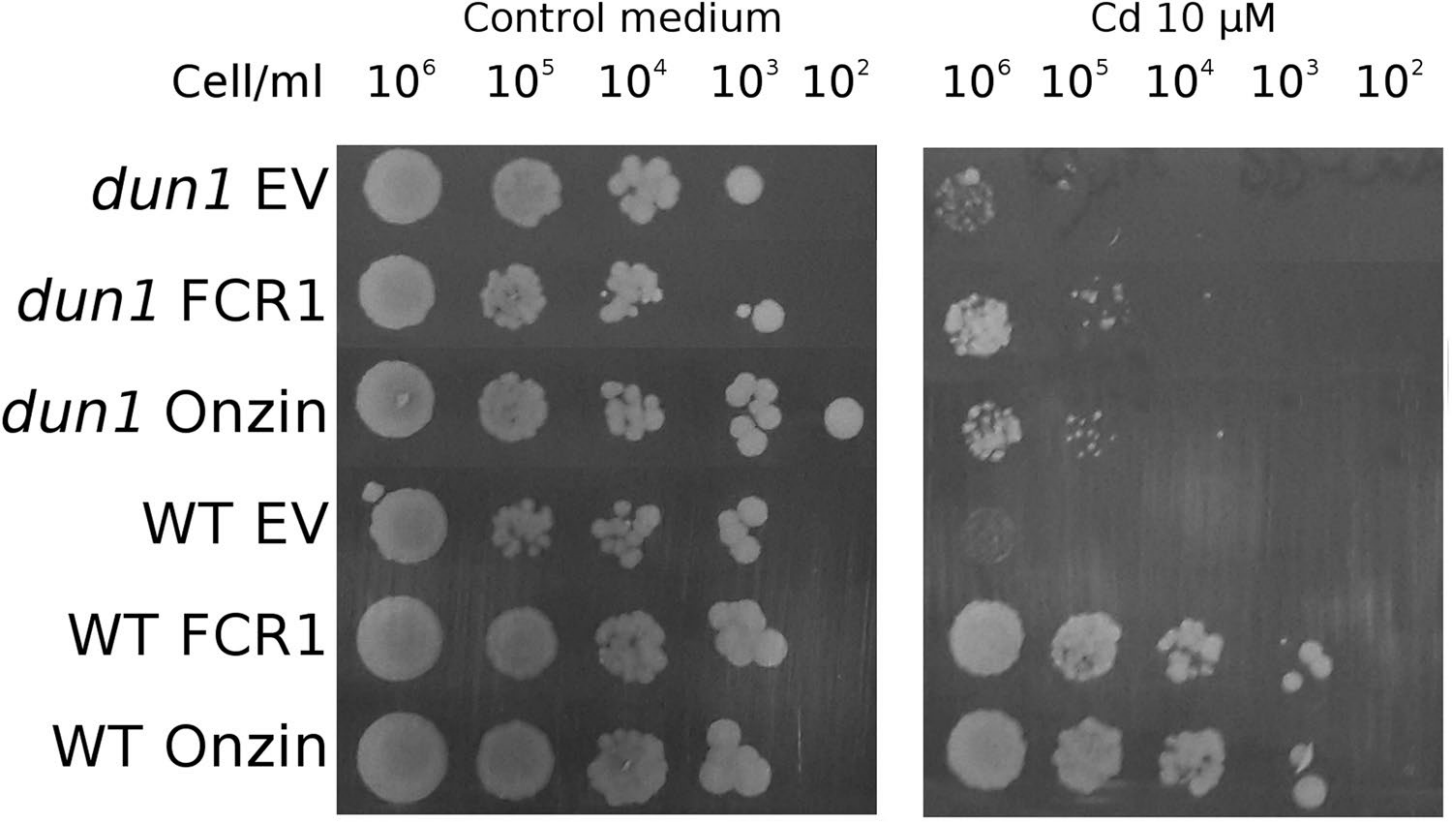
Influence of DUN1 deletion on cadmium tolerance in yeast cells expressing FCR1 and Onzin. Spot dilution assay of wild-type W303 (WT) and *dun1* mutant strains expressing FCR1, Onzin or the empty vector pFL61 (EV). Strains were plated in ten-fold serial dilutions onto YNB-D medium, with or without 10 µM CdSO_4_.

Thus, Dun1p (but not Mec1p) is a central actor in the activation of the DNA damage checkpoint induced by dysfunctions of the ISC assembly machinery^65^. Abbà and colleagues^19^ previously demonstrated that Dun1p is necessary for the cadmium tolerant phenotype of FCR1 in a Mec1p-independent pathway. In this study, we found that Dun1p was also required for the cadmium tolerant phenotype of Onzin-expressing yeast (Fig. 5). Thus, in the presence of nuclear DNA damage caused by cadmium, both PLAC8 proteins seems to activate a Dun1p-dependent DNA damage checkpoint pathway similar to the one described by Pijuan *et al*.^65^ and Sanvisens *et al*.^64^.

### Ancestral functions of PLAC8-containing proteins?

PLAC8 domain containing genes represent a unique gene family^21^ widely distributed in Eukaryotes. The PLAC8 proteins characterized so far are assigned two alternative functions, either in heavy metal resistance or as cell growth regulators. In this work, we showed that Onzin, the reference protein for PLAC8 cell growth regulators^2^, could induce cadmium resistance in *S*. *cerevisiae*, demonstrating that these two functions are not mutually exclusive. Both functions were also found for the rice OsPCR1 that, with different acronyms, was reported as cell growth regulator and able to confer cadmium-resistance^16,66^.

*S*. *cerevisiae* is a simple eukaryotic system where we could likely unravel ancestral functions of PLAC8 proteins. Indeed, the expression of FCR1 and Onzin induced ancient and conserved pathways that play a central role in maintaining DNA integrity and cell growth, the two phenotypes observed in yeast cells exposed to cadmium. In particular, components of the mitochondrial ISC biosynthetic machinery are highly conserved from yeast to humans, and most of them were inherited from prokaryotes^67^.

In the evolution of multicellular organisms, PLAC8 proteins were likely involved in more intricate interacting networks, thus leading to complex organism, tissue, cell and physiological context-dependent phenotypes, such as those observed in mammals. We don’t know how these PLAC8-interacting networks originated and how they evolved. In addition to iron homeostasis, components of the leucine biosynthetic pathway are involved in regulatory functions related to general cell growth and metabolism, and the mitochondrial BCAA transaminase Bat1p is particularly interesting because it shows striking sequence similarity to the mammalian protein Eca39, a known target for c-Myc regulation^68,69^. Myc proteins are involved in cell proliferation and differentiation in vertebrates^70^ but they likely evolved before animal divergence^71^. Although Myc proteins have not been found in yeast, a shortened G1 stage was observed in *bat1* yeast mutants^72^. Thus, in addition to a role in BCAA biosynthesis and ISC translocation, Bat1p is also involved in cell cycle regulation. It is intriguing that Onzin, another known target of c-Myc activity in mammals^2,25^, increased cell survival and growth in the presence of cadmium by modulating the same conserved biosynthetic pathways.

In conclusion, our data clearly demonstrate that two PLAC8 proteins with different described functions in taxonomically distant organisms induced the same phenotype when expressed in *S*. *cerevisiae*, suggesting a common ancestral function. Having identified some processes and pathways regulated by both PLAC8 proteins, the two main functions ascribed to these proteins (i.e. increased cadmium tolerance and regulation of cell proliferation) appear more related, as they could be both linked to leucine and ISC biosynthesis. Although this hypothesis requires further investigations, our results open new perspectives on the role of the PLAC8 protein domain in Eukaryotes and provide guidelines to explore in more details the exact role of the PLAC8 domain in the activation of important biological processes that ensure nuclear DNA integrity during cell division and metal stress responses.

## Methods

### Yeast strains and growth conditions

All yeast strains used in this work are listed in the Supplementary Table S4. EAY1269 wild type strain was kindly provided by Prof. Eric Alani (Cornell University, Ithaca, NY, USA). Yeast strains were grown at 30 °C on Yeast Extract Peptone medium (YP) supplemented with 2% (W/v) glucose (YPD). Yeasts transformed with episomal plasmids were grown at 30 °C on Yeast Nitrogen Base (YNB) medium supplemented with essential amino acids and either 2% (W/v) glucose (YNB-D) or 2% (W/v) galactose (YNB-Gal). All reagents were purchased from Sigma-Aldrich. Yeasts were transformed according to Gietz & Woods^73^. Transformation was confirmed by colony-PCR as described by Sambrook & Russell^74^ with FL1 and FL2 primers (Supplementary Table S5). Strains transformed with pFL61-derived vectors were grown in medium lacking uracil. Yeasts co-transformed with pMS207 vector were grown in medium lacking leucine also. Media and growth conditions for Yeast Two Hybrid were prepared as previously described^19^.

### Protein sequences alignment

The protein alignment was performed using the Phylogeny.fr platform^75^. Sequences were aligned with MUSCLE (v3.8.31) configured for highest accuracy (MUSCLE with default settings).

### Spot dilution assay

Spot dilution assays were used to monitor cell growth at various cadmium concentrations and were conducted as previously described^19^, with the following modifications: for all conditions, overnight yeast cultures were diluted to OD_600_ = 0.1 and cultured at 30 °C until OD_600_ = 0.2. Subsequently, 4 μl of serial dilutions (ranging from 5 × 10^7^ to 5 × 10^2^ cells/ml) of each strain were spotted onto control or cadmium-amended YNB-D medium and incubated at 30 °C for 4 days.

### Determination of cadmium half inhibitory concentration (IC50)

One clone of each EAY1269 yeast strain (transformed with the empty vector or expressing FCR1 or Onzin or Onzin^Δ28–38^) was inoculated in 5 ml YNB-D medium and incubated at 30 °C overnight. The following morning, each pre-inoculum was diluted in fresh YNB-D medium at optical density OD_600_ = 0.1, and poured in 96-well plates. The cultures were amended with increasing concentrations CdSO_4_ (from 0 to 400 µM), incubated at 30 °C and 150 rpm, and the OD_600_ was measured by using a microplate reader (TECAN) after 24 hours. The experiment was independently replicated 3 times, with 5 technical replicates per concentration and per clone (n = 15). IC50 values, representing the Cd concentration causing a 50% of inhibition of the yeast growth, were calculated using the ED50 Plus v1.0 available on line, and previously utilized and validated^76^. Supplementary Fig. S1 shows the distribution of the data from the three experiments, with standard deviation. Different letters indicate statistically different results (p < 0.05). Single data from the three experiments, Shapiro Wilk test for normal distribution, p-values calculated by applying the ANOVA with Tukey as post-hoc test are available in the Supplementary Data S1. All the statistics were performed using PAST^77^.

### Growth curve and cell viability assay

For the growth curve assay, three clones of each EAY1269 yeast strain (transformed with the empty vector or expressing FCR1 or Onzin) were inoculated in 5 ml YNB-D medium and incubated at 30 °C overnight. The following morning, each pre-inoculum was diluted to 50 mL in fresh YNB-D medium at optical density OD_600_ = 0.1, and split into two sterile flasks, one amended with 25 µM cadmium sulphate and the other left unamended. The optical density of cadmium-treated and control cultures was measured after 2, 4, 6, 8, 10, and 24 h of incubation at 30 °C. The data were tested for normal distribution with the Shapiro Wilk test (p-value > 0.05) and the ANOVA was applied with Tukey as post-hoc test. Single data and the calculated p-value for data at 6 and 8 hours are shown in the Supplementary Data S2. Figure 2b shows the result of a single experiment (n = 3) that is representative of three independently repeated experiments, each one based on n = 3 or n = 5 biological replicates, and the raw data obtained after 8 h of incubation from the three experiments are reported in the Supplementary Data S3.

To evaluate the number of viable yeast cells during growth in liquid culture, the number of Colony Forming Units (CFU) was measured by plating 100 μL of the appropriate dilutions (based on the initial OD_600_) onto standard YNB-D medium after 6, 8, 10 and 24 h of growth in control or cadmium-amended liquid YNB-D. Figure 2c reports the distribution of n = 6 biological replicates for the time-points 6, 10 and 24 h and n = 3 biological replicates for the 8 h time-point, obtained in two independent experimental repetitions. Single data, Shapiro Wilk test for normal distribution, p-values calculated by applying the ANOVA with Tukey as post-hoc test are reported in the Supplementary Data S4.

### Onzin coding sequence isolation and expression in yeast

All oligonucleotide sequences are listed in Supplementary Table S5. cDNA corresponding to *Mus musculus* Onzin CDS was synthesized with Qiagen OneStep RT-PCR Kit (Qiagen, Venlo, Netherlands) using total RNA from mouse blood and Not_Onzin1f/Not_Onzin2r primers which carry *NotI* restriction site at the 5′ end. PCR program was as follows: 60 s at 98 °C for 1 cycle; 10 s at 94 °C, 30 s at 60 °C, 40 s at 72 °C for 35 cycles; 10 min at 72 °C for 1 cycle. Amplified DNA was digested with *NotI* restriction enzyme and cloned into the pFL61 Vector. Sequence as well as direction of the inserted gene were assessed by PCR and sequencing using primers FL1 and Not_Onzin2r. This vector was named pFL61-Onzin. The mutant allele Onzin^Δ28−38^ was obtained using a two-round mutagenesis PCR. In the first round, the 5′ end of the sequence was amplified with primers Not_Onzin1f/Onzindel_r while the 3′ end was amplified using primers Onzindel_f/Not_Onzin2r using Thermo Phusion High Fidelity DNA Polymerase (Thermo, Waltham, MA, USA). PCR program was as follows: 30 s at 98 °C for 1 cycle; 10 s at 98 °C, 30 s at 62 °C, 20 s at 72 °C for 35 cycles; 10 min at 72 °C for 1 cycle. In the second round of PCR, the two purified products from the first amplification where used as template for a fusion−PCR using primers Not_Onzin1f /Not_Onzin2r and the same enzyme used before. PCR conditions were as follows: 30 s at 98 °C for 1 cycle; 10 s at 98 °C, 40 s at 62 °C, 20 s at 72 °C for 35 cycles; 10 min at 72 °C. The PCR product was then digested with the restriction enzyme *NotI*, ligated into the plasmid pFL61 previously digested with the same enzyme and cloned into *E*. *coli*. Transformed colonies were screened by colony PCR with primers FL1/Not_Onzin2r and positive plasmids were sequenced with primers FL1 and FL2 to confirm the construct sequence. The pFL61-FCR1 construct has been obtained in a previous work^19^.

### Synthesis of EGFP-tagged Onzin construct and microscopy observations

Construction of C-terminal Enhanced Green Fluorescent Protein (EGFP) tag of Onzin was performed by amplifying the Onzin cDNA with primers HindIII_Onzin_f/XmaI_Onzin_r, carrying *HindIII* and *XmaI* restriction sites and by removing the CDS stop codon. The DNA obtained was then digested and directionally ligated in a previously digested (with the same enzymes) pEGFP-N1 vector and cloned into *E*. *coli*. Colony PCR with primers HindIII_Onzin_f/Not_EGFP_r was used to identify and purify the right construct which was then confirmed by sequencing with the same primers. To express Onzin-EGFP gene in yeast cells, the purified construct was used as template for a second PCR with primers Not_Onzin1f/Not_EGFP_r and the PCR product was digested with *NotI* enzyme, ligated in pFL61 plasmid previously digested with the same enzyme and cloned in *E*. *coli*. Colony PCR using primers Not_Onzin1f/FL2 was used to confirm size and 5′-3′ orientation of the construct. This vector was called pFL61-Onzin-EGFP. The pFL61-FCR1-EGFP construct has been obtained in a previous work^19^.

Yeast cells expressing an inducible tomato protein carrying a nuclear localization signal (NLS)-RFP (plasmid pMS207, courtesy of prof. Maya Schouldiner, Weizmann Institute, Rehovot, Israel) were transformed either with the plasmid carrying the constitutive Onzin-EGFP tagged construct (pFL61-Onzin-EGFP) or with the plasmid carrying the FCR1-EGFP tagged construct. Double transformants were grown overnight with galactose as the sole carbon source (YNB-Gal). The localization of EGFP-tagged and RFP-tagged proteins was observed on a Leica TCS SP2 confocal microscope, using a long-distance 40X water-immersion objective (HCX Apo 0.80). For EGFP visualization, an Argon laser band of 488 nm was used for excitation and the emission window was recorded between 500 and 525 nm, while for the RFP-tagged protein a laser light of 554 nm was used, and an emission window of 581 nm.

### Yeast-Two-Hybrid

The yeast two-hybrid assay was performed using the DupLEX-A yeast system (Origene Technologies, Rockville, MD, USA) as described in previous work^19^. The MmOnzin coding sequence was cloned in frame with the DNA binding domain of LexA into the pEG202 vector using primers Eco_MmOnzin_f/Not_Onzin2r. The C-terminal region of *MLH3* from *Mus musculus* was isolated with Qiagen OneStep RT-PCR Kit (Qiagen, Venlo, The Netherlands) using total RNA from mouse blood and primers Eco_MmMLH3_f/Eco_MmMLH3_r, then cloned downstream in frame with the activator domain of B42 into the pEG202 vector. Sequence and orientation were confirmed by colony-PCR and Sanger sequencing. All the other vectors and strains used in the yeast-two-hybrid assay were generated in previous work^19^.

### Lys^+^ reversion assay

EAY1269 strains (transformed with the empty vector or expressing FCR1 or Onzin) were analyzed for reversion to the Lys^+^ phenotype according to Tran *et al*.^30^. Cells were cultured in YNB-D for 24 hrs at 30 °C and 150 rpm shaking with or without 1 μM CdSO_4_, a concentration that did not influence cell growth in 24 hrs. At the end of the incubation, 100 μl of the appropriate dilution were plated on normal and Lys^−^ dropout medium. Colonies were counted after four days. At least n = 20 independent biological replicates for each strain and condition were analyzed (reported in Table 1). Reversion rates were determined as previously described^78^. Confidence intervals of 95% were determined as described by Dixon & Massey^79^. Each median reversion rate was normalized to the empty vector median rate to calculate the fold increase in mutation rate.

### RNAseq analysis

EAY1269 cells expressing Onzin, FCR1 or transformed with the empty vector (pFL61) were grown in three biological replicates (n = 3), as described for the growth curve, on YNB-D with 25 μM CdSO_4_. After 8hrs, cells were harvested by centrifugation, washed with sterile water and frozen in liquid nitrogen. Total RNA was extracted in a CTAB-based extraction buffer (2% CTAB, 2% PVP, 100 mM Tris-HCl pH 8, 25 mM EDTA, 2 M NaCl, 2% β-mercaptoethanol, 1% (W/v) PVPP). The homogenates were incubated 5 min at 65 °C, extracted twice in chloroform:isoamyl alcohol 24:1 (v/v), precipitated with an equal volume of LiCl 10 M (on-ice over-night precipitation), resuspended in an SSTE buffer (1 M NaCl, 0.5% (W/v) SDS, 10 mM Tris-HCl pH 8, 1 mM EDTA), extracted with phenol:chloroform:isoamyl alcohol 25:24:1 (v/v/v), extracted in chloroform:isoamyl alcohol 24:1 (v/v), precipitated with 100% ethanol (2 hrs, −20 °C), washed in 80% ethanol and resuspended in DEPC-treated water. The quantity and quality of the extracted RNA was evaluated with a 2100 Bioanalyzer (Agilent Technologies, Santa Clara, CA, USA). The mRNA was sequenced by Illumina technology with Hiseq. 2000, raw reads of 50 NTs were firstly assessed for their quality using FastQC and then mapped on *S*. *cerevisiae* S288C genome with Burrows-Wheeler Aligner^80^. Resulting BAM files were processed using SAMtools^81^. Only reads with phred score greater than 15 were considered in the analysis. The genome sequence and annotation files were obtained from SGD database (http://www.yeastgenome.org). The differential gene expression was assessed with DESeq package^82^, retaining only genes with FDR- corrected p-value < 0.1. Genes with read counts equal to zero in all replicates of at least one experimental condition were excluded from the analysis. The fold change was calculated with respect to the empty-vector expressing strain and genes with log_2_ fold-change >1 or <−1 and with adjusted p-value < 0.05 were considered respectively up- or down-regulated by the PLAC8 proteins. Gene Ontology and pathway enrichment were evaluated using DAVID Bioinformatics Resources 6.8 and Saccharomyces Genome Database^83,84^.

### Respiratory activity

Yeast cells were cultured over night at 28 °C in YNB medium supplemented with 0.6% glucose, then cells were treated for 4 hrs with up to 200 µM CdSO_4_. Oxygen consumption rate was measured at 30 °C using a Clark-type oxygen electrode (Oxygraph System Hansatech Instruments England) with 1 mL of air-saturated respiration buffer (0.1 M phthalate–KOH, pH 5.0), 0.5% glucose. The experiment was independently performed twice (n = 2), with one biological replicate per sample.

### Metal content analysis

The EAY1269 yeast strains, transformed with the empty vector or expressing either FCR1 or Onzin, were inoculated in 5 ml YNB-D medium and incubated at 30 °C overnight. The following morning, each pre-inoculum was diluted to 50 mL of the control YNB-D medium, or in the same medium supplemented with 25 μM CdSO_4_. Five different clones were used for each transformant, per each experimental condition (n = 5). Yeast cultures were harvested after 8 h and centrifuged at 4000 rpm for 5 min. Cells were washed three times with 10 mM EDTA in 50 mM Tris–HCl buffer (pH 6.5), and with milli-Q water. Finally, samples were dried at 60 °C for 2 days, and subsequently mineralized with 1 ml HNO_3_ 6 M in a bath at 90 °C for 1 h. After dilution to a final concentration of 1 M HNO_3_, the metal content was determined using Induced Coupled Plasma (ICP-OES Optima 7000 DV, Perkin Elmer). Controls made up of milli-Q water and nitric acid 1 M. Different letters indicate significant differences between samples (p < 0.05). The raw data and exact p-values are reported in the Supplementary Data S5. Normality of data was assessed by Shapiro Wilk test and pairwise differences have been calculated by the ANOVA with Tukey as post-hoc test. When normal distribution was not confirmed, the Mann-Whitney test for differences in the medians was used instead of the ANOVA.

## Supplementary information

Supplementary Figures

Supplementary Data S1

Supplementary Data S2

Supplementary Data S3

Supplementary Data S4

Supplementary Data S5

Supplementary Table S1

Supplementary Table S2

Supplementary Table S3

Supplementary Table S4

Supplementary Table S5

## Data Availability

All relevant data are within the manuscript and its Supporting Information files. The RNAseq data from this publication have been deposited to the SRA database, have been assigned the identifier SRP145576 (BioProject PRJNA471239) and will be available after acceptance.
