# Supplementary material for: Yeast expression of mammalian Onzin and fungal FCR1 suggests ancestral functions of PLAC8 proteins in mitochondrial metabolism and DNA repair
Source: Sci Rep. 2019 Apr 29;9:6629. doi: 10.1038/s41598-019-43136-3 (PMC6488628; doi:10.1038/s41598-019-43136-3)

Title

Yeast expression of mammalian Onzin and fungal FCR1 suggests ancestral functions of PLAC8 proteins in mitochondrial metabolism and DNA repair

Authors

Stefania Daghino, Luigi Di Vietro, Luca Petiti, Elena Martino, Cristina Dallabona, Tiziana Lodi and Silvia Perotto

Figure S1. Half Inhibitory Concentration (IC50) of $CdSO_4$. Yeast expressing FCR1, wild-type Onzin, truncated Onzin$^{\Delta 28\text{-}38}$ or the empty vector pFL61 (EV) were exposed to increasing Cd concentrations and IC50 have been determined. The distribution of the data from three independent experiments is shown in the figure. The square symbol indicates the mean value. The wiskers indicate the minimum and the maximum values. The top and the bottom of the rectangle indicate ± standard deviation, while the central line of the rectangle indicates the 50%. Statistically different results are indicated with different letters (P<0.05 by ANOVA with Tukey as post-hoc test, Shapiro Wilk as normality test).

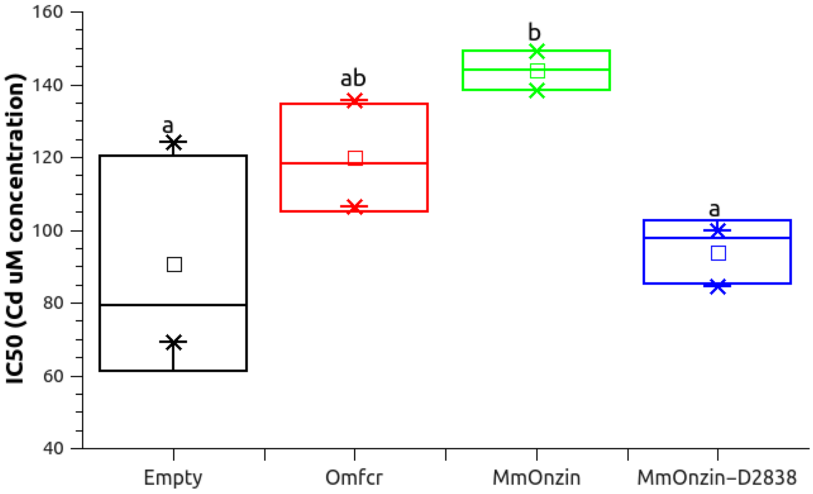

Figure S2. Yeast-Two-Hybrid to investigate FCR1 and Onzin interactions with different Mlh3 proteins. Genes coding for Mlh3p were isolated from *S. cerevisiae* (ScMlh3) from *O. maius* (OmMlh3) and from *Mus musculus* (MmMlh3). Yeasts were plated with ten-fold dilutions onto galactose and β-galactosidase containing-medium lacking leucine. The dark blue color of FCR1/ScMlh3 and FCR1/OmMlh3 colonies indicates a strong protein–protein interaction. Onzin is able to interact with both ScMlh3 and MmMlh3, but the blue color is less intense. No self-activation was observed when cells were co-transformed with a vector expressing an irrelevant protein such as *O. maius* superoxide dismutase (OmSOD1), that was used as negative control. A and B represent two independent replicates of the same experiment.

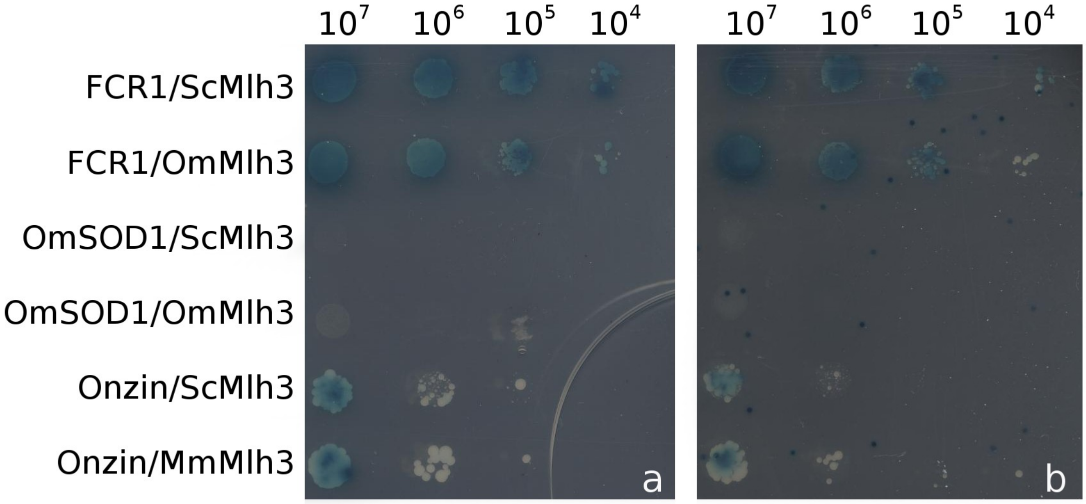

Figure S3. Genes differentially regulated by PLAC8 proteins in the transcriptome of yeast cells exposed to cadmium. The diagram shows the number of genes differentially regulated by Onzin (Onzin-regulated), by FCR1 (FCR1-regulated) or by both proteins (PLAC8 regulated) in yeast cultures grown for 8 h in cadmium-containing medium (25 μM), as compared to the yeast strain transformed with the empty vector.

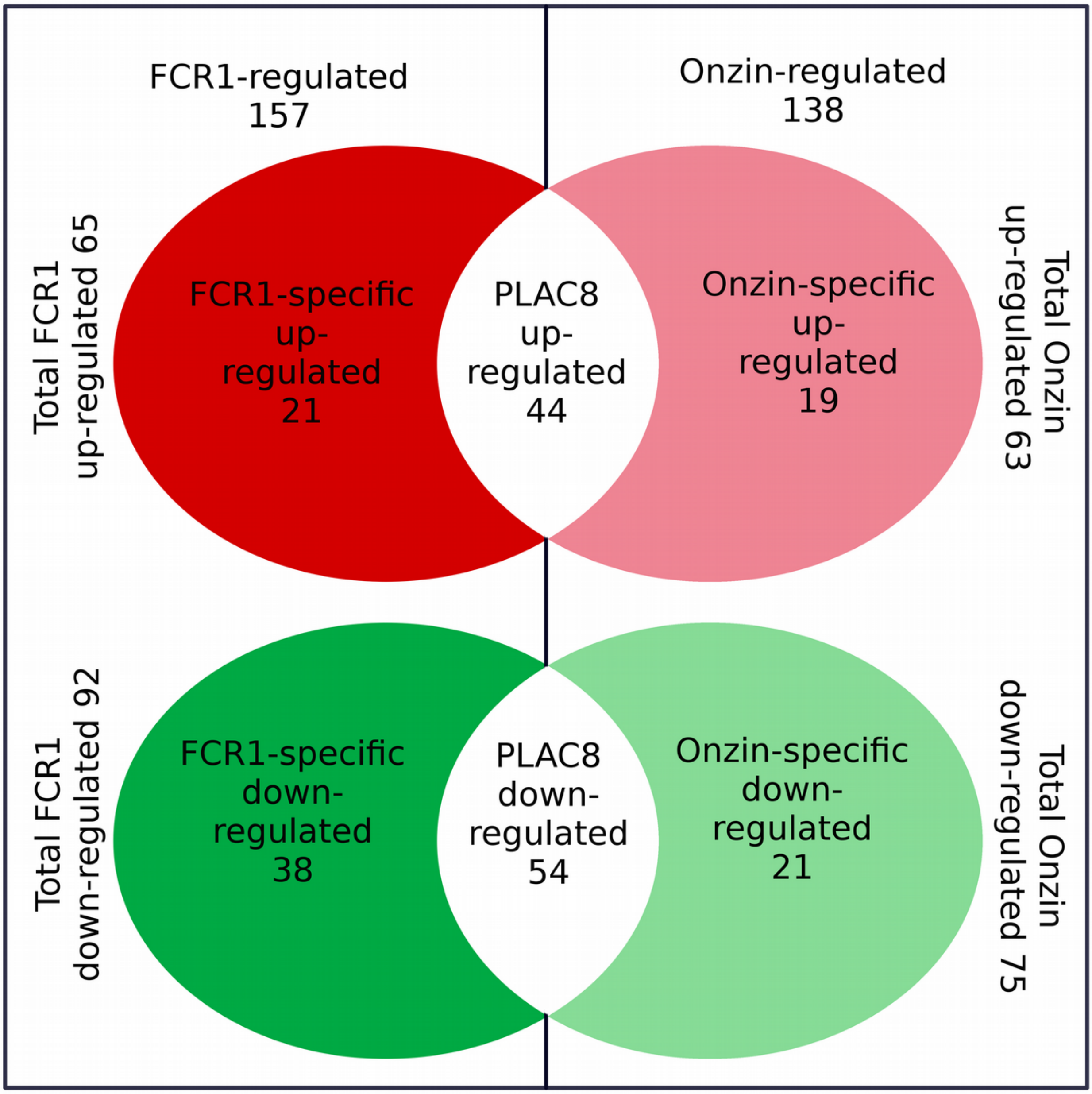

Figure S4. Oxygen consumption rate of yeast strains expressing FCR1, Onzin or the empty vector. Yeast cells were exposed to different $CdSO_4$ concentrations, ranging from 0 to 200 µM. The data from two independent assays are plotted.

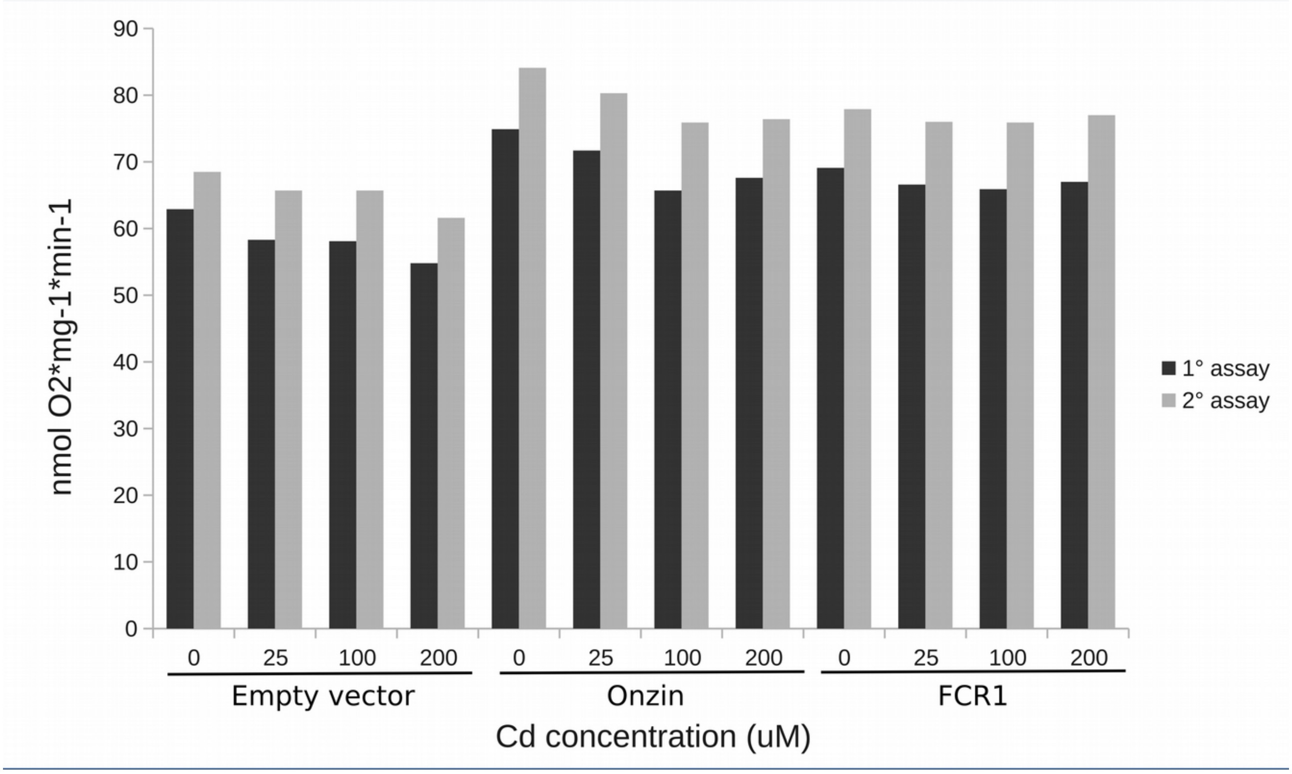

Supplement: Supplementary file 1 — Supplementary Figures [file 41598_2019_43136_MOESM1_ESM.pdf]
